# Identifying and Reducing Remaining Stocks of Rinderpest Virus 

**DOI:** 10.3201/eid2112.150227

**Published:** 2015-12

**Authors:** Keith Hamilton, Dawid Visser, Brian Evans, Bernard Vallat

**Affiliations:** World Organisation for Animal Health, Paris, France

**Keywords:** rinderpest virus, rinderpest material, eradication, global freedom, sequestration, destruction, viruses

## Abstract

In 2011, the world was declared free from rinderpest, one of the most feared and devastating infectious diseases of animals. Rinderpest is the second infectious disease, after smallpox, to have been eradicated. However, potentially infectious rinderpest virus material remains widely disseminated among research and diagnostic facilities across the world and poses a risk for disease recurrence should it be released. Member Countries of the World Organisation for Animal Health and the Food and Agricultural Organization of the United Nations are committed to destroying remaining stocks of infectious material or ensuring that it is stored under international supervision in a limited number of approved facilities. To facilitate this commitment and maintain global freedom from rinderpest, World Organisation for Animal Health Member Countries must report annually on rinderpest material held in their countries. The first official surveys, conducted during 2013–2015, revealed that rinderpest material was stored in an unacceptably high number of facilities and countries.

## Abstract 2

Virus-containing material remains stored in an unacceptably high number of facilities worldwide.

Rinderpest, also known as cattle plague, is a highly contagious viral disease of cattle. Until global freedom from rinderpest was declared in 2011, it was one of the most devastating and feared infectious diseases of animals (*1*). Infection with rinderpest virus (a morbillivirus) led to severe illness and death. Mortality rates in susceptible cattle populations could exceed 90%. Outbreaks have led to food shortages, economic losses, social unrest, and disrupted transport networks in regions where agriculture was dependent on draft cattle (*1*).

It has been suggested that rinderpest originated in central Asia. Over the centuries, the disease swept through Asia and was subsequently introduced into Africa, resulting in “the great African rinderpest pandemic of the 20th century” (*2*,*3*). Apart from an isolated outbreak in Brazil in 1920 and one in Australia in 1923, rinderpest has not affected countries in the Americas or Australia (*4*).

During the 20th century, control efforts became better coordinated and more effective, greatly facilitated by the availability of improved diagnostics and vaccine technologies (*5*). After a concerted international eradication campaign, success was finally achieved at the beginning of the 21st century; global freedom was declared in 2011, a decade after the last reported case of rinderpest had been detected in wildlife in Kenya in 2001 (*6*). After smallpox, rinderpest is the second infectious disease to have been eradicated through the efforts of mankind.

Throughout the eradication campaign, in affected and nonaffected countries, rinderpest material became widely disseminated in diagnostic laboratories, vaccine production facilities, and research institutes. While efforts were focused on eradication, less thought was probably given to what would happen to this material after eradication. In 2015, although natural infections in animals have been eradicated, live rinderpest virus, vaccines, and genetic material remain stored in scientific institutes across the world. 

Today an outbreak of rinderpest could occur only if infectious material held in these laboratories and other institutions were accidentally released into a susceptible animal population or if animals were deliberately infected. The social and economic effects of a recurrence for the international community would be substantial. Vaccination against rinderpest has been prohibited (*7*). Therefore, cattle populations are fully susceptible and infection would spread rapidly if the virus were reintroduced. Recurrence of the disease would seriously damage agricultural economies, would paralyze trade in animals and animal products in affected regions, and would undermine the decades of investment and effort that went into its eradication. Accidental inoculation of cattle with a rinderpest vaccine would also be disruptive because the detection of seropositive animals would lead to suspicion of rinderpest recurrence (*7*). To ensure that rinderpest remains confined to the history books, international efforts are now focused on ensuring that all remaining stocks of infectious material are destroyed or stored safely in a minimum number of approved high-containment facilities.

In 2010, for the purpose of regulating postrinderpest eradication activities, the World Organisation for Animal Health (OIE) and the Food and Agricultural Organization of the United Nations (FAO) Advisory Committee (a specialist body of selected laboratory and rinderpest experts) described potentially infective material (the material that needs to be regulated and safeguarded to prevent a recurrence) as “rinderpest virus–containing material.” The material was defined as follows: rinderpest virus–containing material means field and laboratory strains of rinderpest virus; vaccine strains of rinderpest virus including valid and expired vaccine stocks; tissues, sera, and other clinical material from infected or suspect animals; and diagnostic material containing or encoding live virus. Recombinant morbilliviruses (segmented or nonsegmented) containing unique rinderpest virus nucleic acid or amino acid sequences are considered to be rinderpest virus. Full-length genomic material, including virus RNA and cDNA copies of virus RNA, is considered to be rinderpest virus–containing material. Subgenomic fragments of morbillivirus nucleic acid that are not capable of being incorporated in a replicating morbillivirus or morbillivirus-like virus are not considered as rinderpest virus–containing material (*8*)*.*

Hereafter, we refer to the above-described material as “rinderpest material.” By adopting 3 resolutions (nos. 18, 23, 25), all OIE Member Countries committed to destroying remaining stocks of rinderpest material or ensuring that the material would be stored securely in a minimum number of approved facilities (*8**,**9*) (http://www.oie.int/en/about-us/key-texts/resolutions-and-recommendations/resolutions-adopted-by-the-oie-international-committee/). The OIE and the FAO launched a work program to help Member Countries fulfill this commitment.

To safeguard remaining rinderpest material and to facilitate and monitor its destruction, knowledge of where the material is stored and close monitoring of the status of these stocks are crucial. Updated international standards on rinderpest in the OIE Terrestrial Animal Health Code make it a legal requirement for countries to report annually to the OIE on the nature, whereabouts, and quantity of rinderpest material held in each country (*7*).

During 2013–2014, the OIE conducted the first official survey to identify the precise location of remaining stocks of rinderpest material, and during 2014–2015, the second official survey was conducted. This article summarizes the results from these 2 surveys.

## Methods

The following countries were selected to participate in the survey: 180 OIE Member Countries and Territories as of 2014 (which includes all countries that have a large livestock population) (*10**,**11*), and 2 other non–OIE Member Countries that may potentially have held rinderpest material. When the first survey was initiated in 2014, there were 178 OIE Member Countries; however, 2 additional countries were adopted as OIE Members in May 2014, bringing the total to 180 before the survey was completed. These 2 countries had submitted reports as non-OIE Members in 2014 before their adoption as Members.

Data on the number of countries that had reported an outbreak of rinderpest and the date of the last reported infection were collected from the OIE World Animal Health Information Database (*4*). To maintain confidentiality and to prevent identification of individual countries, the data in this article have been anonymized.

To facilitate reporting, OIE developed a standard questionnaire and a secure electronic system for returning completed questionnaires. A username and unique secure password were issued to the OIE Delegate in each Member Country and the Delegate identified as the responsible person in the National Veterinary Service to oversee completion of the questionnaire. The OIE Delegate is the official OIE representative for an OIE Member Country and is usually the Chief Veterinary Officer or equivalent.

Countries were required to answer an introductory question: “Does your country currently hold rinderpest virus–containing material?” Only 1 of 4 predetermined options could be selected: “yes,” “no,” “unknown,” or “never held rinderpest virus–containing material.” Responders who answered “yes” were asked to provide further information, including details about the nature and quantity of rinderpest virus held, the name and address of the facility where it was held, and the biosafety/biocontainment level of the facility. When 1 of the other 3 options (no, unknown, or never held) was selected, then no further responses were required, and the questionnaire was considered complete.

Countries that reported having rinderpest material were required to provide details about the nature and quantity of material held for the following categories:

- live virus, including field isolates and genetically modified viruses but excluding stocks of approved/registered vaccines;
- vaccine stocks, including seed stocks;
- other potentially infectious materials;
- other noninfectious rinderpest virus–containing materials.

Responders were asked to provide information about rinderpest material currently held, material destroyed during the previous 12 months, and material that had been transferred to or from another institute. Questions also asked whether the institute had conducted any manipulation of rinderpest material in the previous 12 months and whether they intended to destroy material or transfer it to another institute for safer keeping

After responders had submitted the completed questionnaire to the OIE, they still had access to their respective completed questionnaire in a noneditable PDF format. If institutes held a large number of different strains of virus, or types of tissue, then the country could return information in an Excel (Microsoft, Redmond, WA, USA) spreadsheet format.

The OIE Terrestrial Animal Health Code specifies that the deadline for submitting the annual OIE rinderpest report each year is the end of November (*7*). However, because countries were unable to meet this deadline, deadlines for the first 2 surveys were extended until May 26, 2014, and June 11, 2015, respectively. A weekly Excel report was exported from the electronic database for evaluation of information received and to enable the OIE to follow up on erroneous reports and nonresponders. Weekly follow-up with nonresponders included telephone calls and email correspondence. When countries were unable to use the electronic reporting system, they were asked to submit a hard copy (paper) report to the OIE on a template provided. Countries were given 2 opportunities to validate their data: 1) Member Countries that had submitted their report to the OIE were sent a noneditable PDF version of their completed questionnaire and asked to confirm the accuracy of their data; and 2) during the May 2014 and May 2015 OIE General Sessions, all Member Countries were given the complete datasets and a final opportunity for comment.

## Results

For the first survey (completed in 2014), 171 (95%) of 180 OIE Member Countries responded to the survey, and for the second survey (completed in 2015), all 180 OIE Member Countries responded. Additionally, 2 countries that are still (as of September 2015) not OIE Members reported in 2014 but they did not report again in 2015. In 2014, of the 173 countries that responded to the survey, 23 (13.3%) reported holding stocks of rinderpest material; and in 2015, of 180 countries, 24 (13.3%) reported holding stocks of rinderpest material. All countries that reported holding stocks of rinderpest material in 2014 reported still holding rinderpest material in 2015. One country that had reported not holding stocks of rinderpest material in the 2014 survey subsequently discovered that it did hold stocks and reported holding rinderpest material in 2015. For 1 country that reported in both surveys that it held rinderpest material, whether the material (a subgenomic fragment of DNA) constituted the intended meaning of rinderpest material was in doubt. All countries that reported holding stocks of rinderpest material were OIE Member Countries.

Of the 24 countries that reported holding rinderpest material, 1 country reported holding it in 5 institutes in 2014; this country subsequently destroyed all the stocks that were held in 1 institute. Another country reported holding rinderpest material in 2 institutes in 2014 and consolidated the material to 1 institute in 2015. All other countries reported holding the material in only 1 institute. By 2015, a total of 27 institutes in 24 countries reportedly held rinderpest material. In 9 facilities, rinderpest material was stored at Biosafety Level 2 (BSL-2), and in 18 facilities the material was stored at BSL-3 or BSL-4. 

The regions with the greatest number of institutes holding rinderpest material were Asia, Pacific, and Oceania (*9*), followed by Africa (*7*), Europe (*7*), the Americas (*3*), and the Middle East (*1*). OIE regions are described at http://www.oie.int/en/about-us/wo/regional-commissions/.

At least 23 of the 24 countries reporting having rinderpest material held the live virus (including wild strains of virus, vaccine seed virus, and attenuated virus). Because of the questionnaire design, it was not possible to differentiate between vaccine seed strains and packaged and manufactured vaccine, unless this differentiation was specified by the country. Also not provided by some countries was complete information on passage history of virus isolates. By 2015, a total of 22 facilities indicated that they stored vaccine seed virus (which would be classified as live virus). According to data provided in 2015, a total of 7 facilities indicated that they stored manufactured and packaged vaccine.

## Discussion

The annual rinderpest survey serves several purposes that support and facilitate the destruction and safeguarding of remaining stocks of potentially infective rinderpest material.

- It identifies the whereabouts of remaining rinderpest material so that action can be taken to ensure that these stocks are destroyed or stored safely.
- It monitors and evaluates progress of the rinderpest destruction and sequestration program.
- It locates stocks of rinderpest vaccine that could be mobilized in the event of a recurrence of disease.
- Because the whole dataset is shared with all OIE Member Countries, it is hoped that transparency will encourage OIE Member Countries to comply with their commitment to destroy stocks or to store them safely.

As of June 2015, the survey response rate was 100%, indicating that all OIE Member Countries had fulfilled their obligation to report on remaining stocks of rinderpest virus. Four years after the declaration of global freedom, rinderpest material remains stored in at least 27 facilities in 24 countries. Responses indicate that one third of these stocks are stored in facilities equivalent to BSL-2. Considering the potential consequences of a recurrence of rinderpest, this situation represents an unnecessarily high risk.

The data obtained from the surveys may underestimate the real number of facilities holding rinderpest material because there are several potential sources of underreporting. Rinderpest material might be stored in some countries without the knowledge of the reporting authorities, which was confirmed when 1 country submitted a negative report in 2014 and a positive report in 2015. Countries with strong and well-governed official National Veterinary Services should have a system to regulate the shipment, handling, and storage of dangerous pathogens (rinderpest virus is considered a dangerous pathogen for animals). In theory, these systems should identify where stocks of rinderpest virus are being held; however, on a global level, National Veterinary Services are not universally strong. Many countries have a weak regulatory framework, and even in countries with a strong regulatory framework, mistakes occur. Therefore, a country’s Veterinary Services may be unaware of material that is held in facilities outside of their direct jurisdiction, such as in universities or private laboratories. Also, samples containing rinderpest material may have been poorly identified or not included in a laboratory inventory. Pathogen inventories and quality management systems are unlikely to have been in place in all facilities receiving and storing rinderpest virus several decades ago. Archived pathology and surveillance samples collected from animals for reasons other than rinderpest diagnosis may also contain rinderpest virus if these samples were collected in areas where rinderpest was prevalent and stored under conditions suitable for virus survival.

All countries should be encouraged to continue to search for rinderpest material in any places where it may have been held with or without the institute’s knowledge; such places might include laboratories outside the direct control of the official National Veterinary Services, including private laboratories and universities. Institutes that have less contact with National Veterinary Services might even be unaware that rinderpest has been eradicated and unaware of the international commitment to destroy or safeguard remaining stocks. The surprise discovery of smallpox virus at the US National Institutes of Health in 2014 highlights the possibility that unidentified dangerous material may lie in storage unnoticed for years and underscores the need to maintain current and accurate laboratory inventories (*12*).

In addition, no information is available about the viability of live virus for those countries that reported holding stocks of rinderpest material. For rinderpest virus to remain viable during storage over long periods, the material must be continuously kept under suitable conditions. Rinderpest virus is relatively labile, and some institutes with stores of rinderpest have probably experienced power supply disruption, leading to thawing and destruction of the virus. Therefore, some reported stocks of live rinderpest virus might not contain viable virus.

Two countries holding stocks of rinderpest virus have never experienced an outbreak of rinderpest. Because each of these countries has substantial agricultural and veterinary research sectors, it can be assumed that virus was held for research and for preparedness (e.g., diagnostics, vaccine manufacture) purposes. Other countries will have probably kept rinderpest material for the same reasons. However, in a postrinderpest era, the value and justification for maintaining rinderpest material for research are minimal.

The risk for pathogen release from containment laboratories into susceptible animal populations, albeit low, is real. This risk was highlighted in 2007 when a biosecurity breach at a site in the United Kingdom resulted in an outbreak of foot-and-mouth disease among cattle (*13**,**14*). Fortunately, infection was detected early and the source was identified quickly. These actions, combined with an effective response, prevented a wider outbreak, which could have severely hurt the economy (*15*). Tragically, the last case of smallpox was also caused by an escape of the virus from a laboratory (in Birmingham, UK, in 1978), resulting in a human death (*16*). Action must be taken to ensure that a future case of rinderpest does not occur through an avoidable laboratory escape of virus.

On a positive note, 6 institutes in 6 countries had destroyed some rinderpest material during 2013–2015. It is hoped that other countries holding rinderpest material will take similar action.

If countries comply with their commitment to destroy rinderpest material or ensure that it is secured in 1 of the facilities approved by the OIE and FAO, the risk for disease recurrence after a laboratory escape or deliberate release of virus can be substantially reduced. This risk can be further reduced if all known stocks of potentially infective rinderpest material (particularly live virus) worldwide are totally destroyed. To address concerns about the loss of historical data, entire genes of rinderpest virus isolates could be sequenced before destruction—a process commonly referred to as sequence and destroy—and archived with data about the epidemiology and pathology of those viruses. The sequence-and-destroy procedure for rinderpest virus will be used as an additional incentive to encourage scientists to destroy high-risk biological material while retaining academic and historical data that may have research value.

OIE Member Countries should fulfill their international obligation to continue to report to the OIE on an annual basis so that the OIE can monitor and transparently report progress on sequestration and destruction over time. The OIE and FAO have been working with the World Health Organization to apply lessons learned from the smallpox posteradication era to rinderpest. It is hoped that the experience gained during the rinderpest posteradication era will support future programs for eradication of other diseases.
